# Co-designing adult weight management services: a qualitative study exploring barriers, facilitators, and considerations for future commissioning

**DOI:** 10.1186/s12889-024-18031-w

**Published:** 2024-03-12

**Authors:** Rebecca Langford, Rowan Brockman, Jonathan Banks, Russell Jago, Fiona Gillison, Karen Coulman, Theresa Moore, James Nobles

**Affiliations:** 1 The National Institute for Health and Care Research Applied Research Collaboration West (NIHR ARC West), University Hospitals Bristol and Weston NHS Foundation Trust, 9th Floor, Whitefriars, Lewins Mead, BS1 2NT Bristol, UK; 2 Population Health Sciences, Bristol Medical School, University of Bristol, Canynge Hall, 39 Whatley Road, BS8 2PS Bristol, UK; 3 NIHR Bristol Biomedical Research Centre, University Hospitals Bristol and Weston NHS Foundation Trust and University of Bristol, Oakfield House, Oakfield Grove, BS8 2BN Bristol, UK; 4 Centre for Motivation and Health Behaviour Change, Department for Health, University of Bath, BA2 7AY Bath, UK; 5 Population Health Sciences, Bristol Medical School, University of Bristol, 1-5 Whiteladies Road, BS8 1NU Bristol, UK; 6 Health, Nutrition & Environment, Leeds Beckett University, Calverley Building, City Campus, LS1 3HE Leeds, UK

**Keywords:** Co-design, Co-production, Co-creation, Community engagement, Obesity, Weight management, Evaluation, Commissioning, Qualitative, UK

## Abstract

**Background:**

Weight management services have not always benefitted everyone equally. People who live in more deprived areas, racially minoritised communities, those with complex additional needs (e.g., a physical or mental disability), and men are less likely to take part in weight management services. This can subsequently widen health inequalities. One way to counter this is to co-design services with under-served groups to better meet their needs. Using a case study approach, we explored how co-designed adult weight management services were developed, the barriers and facilitators to co-design, and the implications for future commissioning.

**Methods:**

We selected four case studies of adult weight management services in Southwest England where co-design had been planned, representing a range of populations and settings. In each case, we recruited commissioners and providers of the services, and where possible, community members involved in co-design activities. Interviews were conducted online, audio-recorded, transcribed verbatim, and analysed using thematic analysis.

**Results:**

We interviewed 18 participants (8 female; 10 male): seven commissioners, eight providers, and three community members involved in co-designing the services. The case studies used a range of co-design activities (planned and actualised), from light-touch to more in-depth approaches. In two case studies, co-design activities were planned but were not fully implemented due to organisational time or funding constraints. Co-design was viewed positively by participants as a way of creating more appropriate services and better engagement, thus potentially leading to reduced inequalities. Building relationships– with communities, individual community members, and with partner organisations– was critical for successful co-design and took time and effort. Short-term and unpredictable funding often hindered co-design efforts and could damage relationships with communities. Some commissioners raised concerns over the limited evidence for co-design, while others described having to embrace *“a different way of thinking”* when commissioning for co-design.

**Conclusions:**

Co-design is an increasingly popular approach to designing health in services but can be difficult to achieve within traditional funding and commissioning practices. Drawing on our case studies, we present key considerations for those wanting to co-design health services, noting the importance of building strong relationships, creating supportive organisational cultures, and developing the evidence base.

**Supplementary Information:**

The online version contains supplementary material available at 10.1186/s12889-024-18031-w.

## Background

Nearly two-thirds of UK adults are living with overweight or obesity, with higher prevalence among people living in deprived areas, men, certain racially minoritised populations, and adults with learning disabilities [1–3]. Providing direct support to adults who wish to manage their weight is a key national and local government priority [4], as well as that of the health service [5]. This can be done through weight management services which typically include “behaviour change strategies to increase people’s physical activity or decrease inactivity, improve eating behaviour and the quality of a person’s diet” [6]. However, in the past weight management services have had the potential to widen inequalities because those who would benefit the most from support are less likely to engage and most likely to dropout [7].

One possible solution to this problem is to ‘co-design’ weight management services with meaningful input from members of under-served communities to ensure interventions better meet their needs. Co-designing services may help remove barriers to accessing weight management, potentially leading to more equitable engagement and improved health outcomes for service users [8].

Involving community members in developing services has become increasingly popular in recent years. Peer-reviewed articles on co-design and health have increased by 25% per year between 2004 and 2019 [9], with the rate accelerating in the past decade [10]. Dudau and colleagues [11] describe the ‘co-’ paradigm as intuitively appealing, while Filipe et al. [12] describe co-production as a ‘hot topic’ and suggest it has become a mainstream term in UK public policy, governance and research discourse.

There is some evidence to suggest that co-design can positively impact on health outcomes, within research contexts at least. A recent meta-analysis found small to medium effects on a range of social or community-level outcomes, healthcare outcomes, and physical health and health behaviour outcomes, though impact on longer-term outcomes was rarely reported [13]. Other reviews have identified key principles for success in co-design, including systems-based perspectives, embracing creative approaches, focusing on win-win situations for all co-design partners, building on existing skills and interests, and embedding co-design into organisational cultures [13–15].

Several reviews have explored the use of co-design within health services, focusing on acute health care settings [16], chronic disease prevention [10], public health [17], cardiovascular disease [18] and immigrant health [19]. None of these co-design reviews (and few of the studies included within these reviews) focused specifically on adult obesity or weight management services, reflecting a gap in the literature. Within the UK, published literature on co-designed approaches to weight management services is limited. A scoping review of co-designed weight management services is currently underway [20] but only three UK-based studies have been identified [21–23].

The idea for this research project was established through discussions with our local authority and NHS stakeholders. The National Institute for Health and Care Research (NIHR) Applied Research Collaboration (ARC) West brings together stakeholders from across the health and social care systems in the West of England to identify opportunities for applied research. During discussions in 2021, local authority and health service stakeholders (e.g. commissioners and weight management service leads) highlighted that many were working with local community groups to co-design weight management services. Given the lack of UK-specific literature on co-designed weight management services we worked with these local stakeholders to develop a research proposal to examine the utility of co-designed weight management services.

The aims of this study were to:

- Explore how and why co-designed adult weight management services were developed;
- Examine barriers and facilitators to the co-design process; and.
- Identify considerations to support the future commissioning of co-designed services.

## Methods

### Co-design definition

Numerous ‘co-’ terms are used to describe the involvement of local communities and/or populations of interest in the development of services, including ‘co-design’, ‘co-production’, and ‘co-creation’, as well as more general terms such as ‘community engagement’ [8, 10, 24, 25]. Definitions of these terms are inconsistent and contested [25–28]. For this study, ‘co-design’ is viewed as an umbrella term for the involvement of target service users in the planning or development of services.

### Study design

A qualitative case study approach [29] was selected to allow for in-depth exploration of co-design processes in a range of settings and populations.

### Case study selection

Based on our local knowledge and preliminary discussions with our stakeholders, we identified seven possible weight management services from four local authorities (LA) or Integrated Care Boards (ICB) in the region. Further information on these services was gathered through discussion with service leads. From this mapping, and through consultation with our stakeholders, four services were selected to provide a manageable but diverse sample based on their (a) geographical location, (b) planned co-design activities, and (c) intended population (e.g., men, racially minoritised populations.)

Though all four selected services planned to co-design, it became apparent during early conversations with interview participants that these activities had not been fully realised in two of our services. We included these services as they offered valuable insight into the barriers to co-design.

### Participant recruitment

We interviewed three categories of participants in each service. First, local authority or Integrated Care Board commissioners who controlled the funding and commissioned the co-designed weight management services. Second, the providers - staff employed by the local authority, private company or charity who led the co-design process and ran the weight management services. And third, community members who were involved in co-designing the services.

For commissioner and provider interviews, we interviewed all staff who were directly involved in the co-designed weight management services. For ethical and data protection reasons, we were unable to contact community members directly. We asked providers to send out an email invite and information sheet to those who had participated in the co-design process. Four community members responded, though one later declined to be interviewed online.

The study was approved by the Faculty of Health Sciences Research Ethics committee at the University of Bristol (ref: 12,388) and recorded verbal consent was obtained for all participants.

### Data collection

Participants were interviewed via video call by experienced qualitative researchers (RB or RL) between January and June 2023. We developed a broad topic guide to cover key questions while allowing flexibility within interviews (supplementary file 1). Commissioners and providers interviews focused on the background to the project, why a co-design approach was chosen, the co-design process and engagement, and reflections on the co-design process. For community members, interviews focused on their experience of co-designing the service and reflections on this process. Interviews lasted 33–80 min, were audio-recorded, stored securely, and transcribed verbatim. Community members were offered a £20 voucher for participating; commissioners and providers were not.

### Data analysis

Transcripts were checked and anonymised before analysis. RB and RL read through the same three transcripts, independently noting potential codes which were then developed through discussion into a coding framework (see supplementary file 2). Codes were developed deductively (from interview questions) and inductively (from participants’ responses). This framework was used to code transcripts, with modifications/additions made where necessary. RB and RL coded separate transcripts using NVivo (release 1.7.1), with all coding checked by the other. Codes and sub-codes were summarised, scrutinised and revised, with links and connections between codes identified, mapped and discussed in the process of creating our analytical themes [30, 31].

### Public involvement

We recruited three public contributors living with obesity via Obesity Voices as part of our research management team who offered a lived-experience perspective on the research. As part of this team, they contributed to the oversight of the project, provided feedback on analyses, and contributed to study outputs. They were later joined by a further three public contributors who provided input into study outputs and dissemination plans. These participants were paid for their time. An induction to the study and their role was provided by RL and RB, and ongoing support was provided by the Patient and Public Involvement co-ordinators at Obesity Voices and ARC West.

## Results

We interviewed 18 (8 female; 10 male) participants in total; seven commissioners, eight service providers, and three community members (Table 1). Two services (Healthy Choices, Active People) did not conduct their co-design activities as planned (for reasons discussed below) meaning there were no community members to interview. Requests to the FitnFun providers to facilitate recruitment of community members involved in the co-design received no response. An overview of each case study is provided Table 2.

**Table 1:** List of participants

Project Name	Healthy Choices (HC)	FitnFun (FnF)	Active People (AP)	Men’s Project (MP)
Area	Local Authority 1	Local Authority 1	Local Authority 2	Integrated Care Board 1
Intended target population	Racially minoritised populations	Adults with mild learning disabilities	High deprivation & racially minoritised populations	Men
Commissioners (C)	3 (2 F, 1 M)		2 (2 F)	2 (2 M)
Providers (P)	1 (F)	1 (F)	4 (1 F, 3 M)	2 (2 M)
Community members (CM)	0	N/A	N/A	3 (3 M)

Numbers indicate number of interviews completed. F = Female, M = Male. N/A = Not Applicable: In two studies co-design activities were not completed meaning there were no community members to interview

**Table 2:** Case study details

Case Study Name	Details
Healthy Choices	**Target population**: Racially minoritised communities. **Background**: Local data suggested uptake of universally commissioned services like Slimming World was low among racially minoritised communities. Commissioners were aware of research evidence suggesting “peer support” interventions were effective and may work well for weight management, and wanted to use this approach to increase uptake and engagement in these communities. The plan was to identify a peer mentor to run the service, working with their community to adapt the course content and activities to local needs. However, the public health team were unable to identify, recruit and train a peer mentor to run the service within the time available. Instead, the course ran using local authority health promotion officers, rather than a peer mentor. Time constraints also limited their ability to effectively recruit racially minoritised participants. Participants were therefore recruited from the weight management services waiting list: predominantly white middle-aged women, who did not want to attend more traditional weight management services. **Co-design activities**: No specific co-design used in determining the overall service due to time constraints. However, there was a focus on tailoring the weight management service to the needs of the individual group members as the service went on. **Final service**: A 12-week service, based on Cancer Research UK’s Ten Top Tips took place at two community settings in central LA1, focusing on an individually-tailored lifestyle approach.
FitnFun	**Target population**: Adults with mild learning disabilities. **Background**: Adults with learning disabilities are more likely than their non-disabled peers to be living with overweight or obesity [3]. Based on the national Change4Life Healthier Families campaign, LA1 had previously created their own adult weight management service, developing an intervention booklet which guided sessions and activities over a series of weeks. With some additional government funding available, the service provider (P14_FnF) suggested they adapt this service for adults with learning disabilities, whom she noted often had poor diets and lacked exercise. **Co-design activities**: LA1 partnered with a local learning disability day centre and advocacy group, with whom they had pre-existing relationships, to conduct the co-design activities. Adults with learning disabilities attending a local day centre were asked to participate in a one-off co-design session. Participants were shown the original healthy weight booklet and discussed what they liked/disliked about it and how it could be modified to suit their needs. Support staff from the day centre and a disability advocacy group also gave their views. Changes made included simplifying the language and removing some of the pictures to avoid distraction, but keeping the vibrant colours. Some physical activities were adapted so they were suitable for wheelchair users, while some food suggestions were adapted to use cheaper ingredients. Feedback from participants included an emphasis on making it “fun” and maintaining a positive body image. The overall service was also tailored to the local setting e.g., referencing local services and including a trip to a local supermarket. **Final service**: A 12-week weight management intervention using a guidance booklet, based on the original healthy weight service, but adapted for use with adults with a mild learning disability. The original service was 10 weeks long, but FitnFun extended this to 12 weeks to allow more time to cover key concepts. The first six sessions included a theory element (e.g., the importance of healthy eating and exercise) and a physically-active game. Participants were encouraged to set personal goals and suggest ideas for what they wanted to cover in later weeks. The second half of service re-visited previous content to reinforce learning and included visits to local supermarkets and taster physical activity sessions at local clubs.
Active People	**Target population**: Racially minoritised populations and areas of high deprivation. **Background**: Active People is a UK-based weight management healthy lifestyles organisation contracted by LA2 to deliver community-based weight management services to both adults and children. Alongside providing weight-management services, Active People were commissioned by LA2 to gather local “insights” with a view to co-producing locally-relevant weight management services. This included extensive community development with local organisations, key stakeholders and members of the public, to map out existing services and build relationships within particular communities within LA2. Active People also employed community members (like P11_AP) from target communities to talk with local people and gather “insights” into what a successful weight management service would look like, though initial conversations were often framed around the concept of “good health” rather than weight per se. This work was building towards specific events with the community members where weight management services would be co-designed. However, government funding was pulled before Active People had the chance to implement these activities. **Co-design activities**: A framework for co-design was created but not fully implemented, due to funding cuts. The planned activities involved four ‘stations’ using the analogy of a kitchen and building a recipe together: 1. What’s in your cupboard? *(What’s in the community already? What can be built on?);* 2. What’s on your shopping list? *(What or who else is needed to create this?);* 3. Method *(How should we make this happen?);* 4. Reflection. *(Are we happy with this? Do we need to change anything?)* **Final service**: Active People offer a range of online or in-person services. Most are 12-week services which are tailored to different audiences including adults, families, people with disabilities and adolescents. No co-design activities took place during this period to modify the service formally, but participants suggested there may have been some minimal changes based on the feedback they were received from their “insights” work.
The Men’s project	**Target population: Men** **Background**: Commissioners at ICB1 had a remit around upstream prevention, focusing on the wider determinants of health and reducing inequalities. Local service data suggested men were not accessing commissioned weight management services like Weight Watchers, which are often perceived by men as not appropriate or accessible [32–34]. Commissioners wanted to create something that would address this unmet need but were aware the NHS label could be off-putting. Commissioners were already aware of a well-respected local charity whose goal was to create social connection through sharing food. Wanting to try something different, they approached the charity and asked them to partner with them in co-designing this service. **Co-design activities**: Co-design activities occurred in both the planning and implementation stages of the project using what the commissioner (C10_MP) called a “social design” approach and drawing on the Design Council’s paper on co-creating health services [35]. An initial informal survey was circulated via the charity’s local social media to ask men their views on health and wellbeing. This led to a series of focus groups to explore what the men wanted to focus on, as well as practical issues such as time of day or group size. Commissioners and providers used this information to create a framework for the service, which was presented to men at an open evening for further feedback and discussion. The service was run successively with two cohorts of 10–12 men, with further co-design occurring throughout: providers provided a loose framework for the initial meeting, but the content and structure of sessions were largely directed by the men thereafter. **Final service**: Six-week peer support group for men (though many participants continued meeting after the service had ended). The service involved men coming together to talk about their mental and physical health, while participating in a group activity often based around the sharing of food. The premise was that increased social connection built resilience and enabled men to talk about their health concerns, which may or may not include weight management. Conversations were loosely structured around principles of health coaching and motivational interviewing, to enable participants to set goals for themselves, but the remit was open and collaborative.

Project names are pseudonyms. Participant IDs indicate category of participant (C = Commissioner; P = Provider; CM = Community Member), interview number, and associated project acronym (Healthy Choices = HC; FitnFun = FnF; Active People = AP; Men’s Project = MP).

We identified five analytical themes, described below, and summarised in Table 3.

**Table 3:** Summary of themes

Theme name	Summary
Perspectives on co-design	• Participants used different terms to describe community involvement– co-design, co-production, co-creation– reflecting different levels of involvement • Co-design was viewed positively by all participants • Co-design was thought to create more suitable services, increase engagement, and potentially reduce inequalities
Building relationships for co-design	• Building relationships with communities was critical for co-design • This took time and could not be rushed, especially in communities where trust had previously been damaged through lack of action • Case studies demonstrated different ways of building relationships with communities e.g., partnering with a local charity, commissioning a private provider, or using in-house resources and connections
Funding for co-design: opportunities and harms	• Funding for co-design is often short-term and ad hoc • Opportunistic funding pots can allow for innovative approaches to be tried • However, small amounts of funding and short time frames can hinder co-design plans • Relationships with communities can be damaged if funding is cut
Evidence, effectiveness and evaluation	• Commissioners recognised co-design lacks a strong evidence base • Tight budgets can favour ‘evidence-based’ programmes over co-designed approaches where effectiveness is unknown • Co-design can be hard to evaluate using traditional approaches and pre-specified outcomes measures
Commissioning for ‘true’ co-design	• Co-design may not easily fit with current commissioning practices • Commissioning for co-design may require a *“a different way of thinking”* • Co-design involves a level of uncertainty requiring commissioners to be “brave” and embrace the possibility of failure • Facilitating true co-design requires working with partners who share your vision and values

### Perspectives on co-design

Participants used a range of ‘co-’ words to describe their activities, with word choice reflecting the intensity of involvement. Commissioners and providers from the Healthy Choices and FitnFun projects were more likely to use the term ‘co-design’ and generally described lower levels of community involvement. Active People and The Men’s Project reflected more in-depth levels of community involvement and preferred the terms ‘co-production’ and ‘co-creation’, respectively.

The FitnFun service sought to engage and consult with adults with learning disabilities to increase the relevance and appeal of the course materials and ensure content was locally tailored. The primary aim was to adapt an existing local service rather than co-design a new one.

For Healthy Choices, the role of co-design was less clear. Though originally intended to be run by peer mentors selected from the local community, it ended up being run by local authority staff, due to time and funding constraints. Based on a pre-existing programme, staff followed the published course content but encouraged participants to identify what they wanted to discuss and *“how they wanted to do it”* (P05_HC). Whether this constituted *“co-design”* was questioned by one of the commissioners who acknowledged the “*peer support element”* was meant to be the *“defining characteristic of that piece of work,”* though she added being *“flexible to adapt to the needs of the group and the insight collected along the way”* was also important (C09_HC).

Active People staff were very intentional with their use of terminology, querying during the interview what researchers meant by “co-design” and differentiating it from their “co-production” approach:

*I don’t think [co-design and co-production] mean the same thing. Co-design implies that you’re sitting down with someone and you’re writing something together… Whereas co-production feels like a collaborative effort that doesn’t have a design in mind, you’re just producing something together.* (P08_AP)

The Active People staff had carefully considered what different terms meant and contrasted their approach– built on trust and empowering local communities– with lower levels of involvement such as *“public and patient involvement… getting people to talk about things, like what’s your feedback on this?”* (P03_AP).

Within the Men’s Project, the commissioner preferred the terms *“co-creation”* or *“co-production”* which she felt recognised the *“depth of the involvement of the individuals, their ability to truly influence what’s happening”* (C10_MP). For her, this collaboration involved more than “*one-off bits of consultation… where there’s no context or relationship*” and encompassed the entire process involving people in *“defining the problem, thinking about solutions, enacting those solutions, defining how you measure it.”*

Participants across all projects spoke positively about the potential benefits of co-designing weight management services with community members. Commissioners and providers recognised current services often failed to meet the needs of certain, often marginalised, groups and that a different approach was needed. Directly involving the intended populations in designing a more suitable service made sense:

*If you’re designing for a less engaged group that’s not represented in the data, where there isn’t literature out there that’s easily translated into service, it just makes sense to pay a lot of attention to working with that group, to understand that problem, and design something for them.* (C10_MP)

Participants suggested co-design could make services more effective. They believed it could help reach the *“people we’re not reaching”* (thus potentially reducing inequalities) and ensure services *“fit the priorities of the [intended] population rather than our agenda”* (C12_MP). On a pragmatic note, co-design helped ensure content was appropriate to the target audience. For example, the FitnFun commissioner recognised their original materials would have been *“too wordy, too complicated”* for adults with learning disabilities, while the provider added:

*Without that user input, we could’ve got [the service] completely wrong [because] guys with [learning disabilities] see things in a completely different way.* (P14_FNF)

Other participants suggested co-design could lead to better engagement because people were more likely to *“stick to”* (P11_AP) something they had helped design. The Men’s Project commissioner (C10_MP) noted the co-design process created *“buy-in”* because the men felt they had *“skin in the game”*, while one of the community members noted:

*If you tell people they’re in charge of designing it, they’re much more likely to remain engaged because if they feel there are flaws or things that could be done, they’ve got the voice.* (CM16_MP)

Though commissioners and providers identified many benefits of co-design, they also acknowledged it was a challenging approach. Co-design took more time, more effort, and was described as *“hard”, “complicated”* or bluntly “really f***ing difficult.” Consequently, despite it being seen as *“the best thing”* to do, it was often not fully realised *“because it’s simplest not to”* (C06_HC).

### Building relationships for co-design

Building strong relationships with communities was critical for co-design. Across all projects, participants were clear co-design was not something that could just happen; it took commitment and patience to establish the trust needed to work with local communities. Creating relationships required a “*softly, softly approach”* (C06_HC) and couldn’t be forced or rushed.

*You can’t just go in and “do” co-production…It takes groundwork. You have to build all these relationships in to do it well.* (P02_AP)

Commissioners and providers talked about building relationships based on the trust established by *“integrity, commitment, transparency”* (P03_AP). Relationships also needed to be on-going and meaningful, and importantly, followed up with action. Participants recognised the damage done when communities were over-consulted, over-promised and left with little to show for it. Re-establishing trust after such experiences was challenging:

*Communities that have been hit by the “hit-and-run”, people saying we’re going to do this… then a month later [they’re] gone. Then that trust might take four, six months, a year to build.* (P03_AP)

Active People and the Men’s Project commissioners both placed particular emphasis on building relationships but approached this in different ways. Active People spent over a year building trust and establishing relationships with local communities, through the community development work of their staff (e.g., P02_AP), as well as the “insights” gathered by local people (like P11_AP) employed by Active People in target neighbourhoods. Commissioners for the Men’s Project took a different approach. Recognising the NHS label could be off-putting (a *“sickness service”*), they chose to partner with a well-respected local charity, capitalising on the goodwill and trust already established by this voluntary sector organisation.

FitnFun commissioners and providers had existing relationships with a local learning disability day centre, so found it relatively straight forward to engage with their intended audience. By contrast, it was notable that the Healthy Choices staff originally wished to focus on racially minoritised communities but, lacking pre-existing relationships, they failed to recruit a peer mentor within the time available.

### Funding for codesign: opportunities and harms

Funding for co-design was frequently raised by commissioners and providers. Building the necessary relationships to facilitate co-design took time and thus incurred costs. However, participants often described a situation of short-term pockets of funding needing to be spent swiftly, creating a disconnected patchwork of small projects. As one of the Healthy Choices commissioners explained,

*They send you money and you’ve got two months to use it… It’s just too quick… it doesn’t allow… that programme development.”* (C06_HC).

There were some potential benefits to these opportunistic funding pots. Projects commissioned by the ICB were largely funded by *“one-off pots of monies [and] underspends”* (C12_MP). Because this money was not ear-marked for specific activities staff could focus on ‘innovation’ and novel approaches, such as partnering with a charity to co-design a new service. Similarly, both Healthy Choices and FitnFun commissioners noted having extra funding allowed them to try something new:

*Any other time it would be difficult in the current financial context to put on an additional intervention… because we had the additional funding, we had the flexibility to try something new.* (C04_FnF)

However, participants also acknowledged short-term funding hindered the co-design process. The FitnFun provider explained they were unable to do as much co-design as they wished because *“we only had 2½ months to do everything”* (P14_FnF). Similarly, the Healthy Choices provider (P05_HC) felt their project was *“rushed”*, meaning they couldn’t recruit a peer mentor or effectively target recruitment towards racially minoritised communities.

The Active People project also illustrated the harms arising from an unpredictable funding landscape. The local commissioner explained she was *“loathe to accept”* the initial offer of one year’s funding for fear of raising expectations. Despite then being promised three years of additional funding, this was pulled at short notice leaving those involved *“truly sad”* (P02_AP), *“disappointed”* (P01_AP) and *“disillusioned”* (P07_AP). With no follow-on funding, the planned “co-production” work was halted, with an Active People staff member explaining their reasoning:

*“Working towards co-production, a lot of it is about trust. We’re about to get people in a room, people we trust, people who trust us, [and] we’re told there’s no more funding. So, do you continue [the co-production] and then say, ‘By the way, after July you’ll never see us again, so all this work, all your time, all your commitment is going to be for nothing’?* (P02_AP)

### Evidence, effectiveness and evaluation

For commissioners in particular, the issue of evidence and effectiveness was important. Commissioners talked about wanting to draw on theory and evidence to underpin their projects but acknowledged *“there’s not loads of evidence out there”* for co-design (C07_AP). How comfortable commissioners were with this lack of evidence appeared to vary. The Active People commissioner (C07_AP) felt there was a need to try something different, recognising the current way of doing things was not working. Seeing how a near-by local authority had worked with the Active People company gave her confidence to try a co-design approach, but noted it required *“faith… and commitment… [because] you haven’t got all the evidence base”.* Similarly, The Men’s Project commissioner explained their focus was on trying something new:

*This was an experiment. We didn’t really come to it with a ‘did it work?’ type paradigm. We came to it with a learning paradigm.* (C10_MP)

By contrast, commissioners for Healthy Choices and FitnFun appeared less comfortable with the lack of evidence, finding this challenging to square with responsible public spending. While the Men’s Project provider felt co-designed services could potentially *“cost less money because it’s designed by the people who are going to use it”* (P13_MP), the Healthy Choices commissioner felt co-design was potentially *“more expensive and time consuming”* (C06_HC) than *“off-the-shelf”* commercial weight loss services that could be commissioned. In the context of tight budgets, they would fund these commercial services: they had *“robust RCT evidence”* and were cost-effective. But there was uncertainty around the co-designed projects, with one commissioner questioning where money would be best spent:

*Are we better spending a huge amount on one* [co-designed] *group and we don’t know how effective it is, compared to less on a wider group where we know the results are reasonable?* (C06_HC)

Co-design and evidence-based practice appeared to sit less comfortably together for these commissioners. When asked about the culture around co-design within his local authority, one commissioner appeared to suggest tension between the two:

*There will be some people who will be evidenced-based through and through but there will be some people that are always thinking of the end service user first.* (C04_FnF)

For these commissioners, there was a clear need to develop *“a really good evidence base for this, otherwise we could show we’re just wasting money”* (C06_HC). One wanted to know whether *“co-designed weight management services are more likely to be successful than [commercial programmes] where you know exactly what you’re going to get?”* (C09_HC). She was also keen to see what other local authorities were doing and how they could learn from them.

However, all commissioners suggested building the evidence base for co-design was hard. The often-short-term nature of funding (with tight spending deadlines) made evaluation challenging. As the FitnFun commission noted, determining effectiveness required *“clear evaluation methodology and we just don’t have the resource to do that”* (C04_FnF).

But there was also a bigger question of what could or should be measured. The Men’s Project, for example, was funded as a weight management project but, being fully *“co-created”*, the men determined its focus. Their priorities were more about loneliness, mental health and building community, than weight loss. As one local participant explained, *“I didn’t join because I thought it was about health, I joined because I thought it was about connection”* (CM15_MP). Indeed, the idea of a weight management group would have actively put off some of these men. Attempting to evaluate it in terms of kilograms lost therefore did not make sense. The Men’s Project commissioner acknowledged this co-created approach was challenging in a *“system geared towards understanding value in a certain way”* (C12_MP), as did one of the local participants:

*It must be very hard for anybody who wants to concretise the benefit in a way which doesn’t feel very amorphous and a bit airy fairy.* (CM15_MP)

Nonetheless, all Men’s Project interviewees were strongly in favour of co-creating the project outcomes. Weight was viewed as a complex issue that could not be separated from other aspects of people’s complicated lives. One of the Men’s Project providers talked about *“root causes”* and recognised *“loneliness might be a reason why someone eats for comfort”* (P18_MP). Men’s Project participants recognised the need to work further upstream and felt this approach could yield benefits in the future. One of the commissioners felt the project created *“the context that enables people to make choices around their weight at a later date”* (C12_MP), a perspective which was confirmed by one of the participants: *“If you want to look purely at health and wellbeing benefits, there are people now doing activities they didn’t used to do”* (CM17_MP.) This level of ownership within the community represented true power-sharing, but nonetheless posed a challenge in demonstrating its value:

*Would I say it was a success? Yes, but that’s taking new perspective on what success is. If I presented this to a board of clinicians or NHS England, would they say it was successful? I don’t know!* (C12_MP)

### Commissioning for ‘true’ co-design

Active People and the Men’s Project participants talked at length about commissioning, noting current approaches often hindered co-design. Commissioning briefs were often highly prescriptive and focused on *“Key Performance Indicators, bums on seats, people through the door”* (P03_AP). Co-design however required much more flexibility, which posed challenges:

*You’re co-creating something, you don’t know what it’s going to be. But you have set a contract for it… a specification. Otherwise, your colleagues will say… ‘What’s this money for?’* (C10_MP).

Commissioning for ‘true’ co-design required *“a different way of thinking”* (C07_MP), a *“change in mindset and culture”* (C01_MP), a *“paradigm shift”* (C12_MP). This shift was neatly summarised by one of the Men’s Project staff:

*Too often people commission* ***the*** *results… we think this is what needs to happen and we’re going to commission someone to make that happen. Whereas co-design allows you to commission* ***for*** *results… saying here’s the money, this is the framework, let’s see what happens.* (P13_MP)

It also required something else: *“courage”* (P03_AP) and a willingness to embrace uncertainty. Participants talked about having to get comfortable with not always knowing what would happen:

*As a commissioner you think you know what you want to come out of it, and it might not be that. If you’re truly going to do co-creation… it demands flexibility and openness… which is not a traditional commissioner role, allowing for that uncertainty and emergence.* (C10_MP)

They acknowledged this could at times be uncomfortable. A Men’s Project staff member described the uncertainty as *“a bit scary”* (P18_MP), while the commissioner talked about needing resilience to stick to this approach within *“a system that wants things in a certain way”* (C12_MP). Similarly, one of the Active People staff noted there is no *“IKEA”* toolkit for *“coproduction”* and that it involves *“lots of ups and downs”* (P03_AP).

Truly collaborative co-design was therefore perceived as a risky undertaking given the lack of evidence; it could only happen within an environment that embraced *“[possible] failure… as a great opportunity for learning”* (P08_AP), something noted as uncommon in political environments like local authorities. As both a staff member and community member of the Men’s Project noted, for co-design to work, people and organisations had to be brave and *“trust the process”* (P18_MP and CM15_MP).

Participants described how this new approach to commissioning meant it was important to find the right people to work with, who shared your vision and values: as one commissioner noted, it was about finding the “*right people… the right ethos.”* (C01_AP). The approach taken by Active People– with its emphasis on *“test and learn”* and *“insights”*– meant commissioners felt they were a natural fit for this work. Similarly, the ICB commissioners felt it was important to find the right partner organisation:

*We’d seen the great work they were doing around food, so it made them an obvious choice… We wanted an organisation who was really connected into community.* (C12_MP)

This worked the other way round too. One of the Active People staff members explained *“[we put] stuff out into the world about what we believed [about co-production]… and people who believe the same thing found us*” (P08_AP). Similarly, the Men’s Project staff member explained *“we hold our [organisational] values very strong”* and that they had carefully considered if the proposed ICB project would fit with their *“mission”* (P13_MP). While initially sceptical about a weight management project, they were drawn to the co-design element, allowing the men to create what was important to them. Similarly, local men appeared to get involved because *“they bought into the [charity] and its values… and were confident it would be a good thing to get involved in”* (C10_MP).

## Discussion

Using a case study approach, this study explored the process of co-designing adult weight management services for traditionally under-served communities, representing a range of approaches to community involvement [36]: from light-touch consultation or engagement activities used in Healthy Choices and FitnFun to revise or adapt existing services; to more in-depth “co-production” or “co-creation” approaches employed by Active People and the Men’s Project.

While co-design was viewed positively by participants as a means of increasing engagement and reducing inequalities, it also presented challenges. Two projects were unable to fully implement their planned co-design activities, highlighting important barriers to co-design such as organisational time constraints and the often-short-term nature of funding. Commissioning for ‘true’ co-design with high levels of community involvement required a shift in mindset, relinquishing control, and embracing a level of uncertainty. This could, however, conflict with other principles such as evidence-based practice and responsible stewardship of public money in the context of tight budgets.

Based on our findings, we highlight in Table 4 some key considerations for those wanting to co-design health services. Broadly, these suggest focusing on building relationships (considerations 1–3), creating supportive organisational cultures (considerations 4–6), and developing the evidence base (consideration 7).

**Table 4:** Considerations for co-design

**1. Consider how best to connect with communities** Partnering with well-respected local voluntary organisations, commissioning private companies, or using in-house resources to build relationship are all options, each with their own advantages and disadvantages.
**2. Spend time with partners developing shared values** Spend time early on discussing with potential partners your values, building a shared vision for co-design and what you hope to achieve.
**3. Build strong relationships** Establishing trust, rapport and credibility with communities is an essential part of co-design: it will take time and cannot be rushed. This may be especially true with traditionally under-served populations.
**4. Get comfortable with uncertainty** Co-design inherently involves a level of uncertainty as stakeholders may view issues in different ways. Co-design should focus on outcomes deemed most important to community members.
**5. Reflect on commissioning approaches** Traditional commissioning approaches may not fit easily with co-design. Consider how you might commission *for* service (to produce longer-term ‘value’) rather than *the* service (focusing on short-term, pre-specified outcomes).
**6. Think about the long game** Consider the sustainability of the work. Short-term or unreliable funding hinders meaningful co-design and can damage relationships with communities.
7. **Build the evidence base** Use evaluation approaches that can adequately capture the often subtle, long-term, and upstream impacts of co-design. Share the successes and the failures.

### Build relationships

Across all four case studies– regardless of depth of involvement– participants recognised it required substantial effort to build the trust and relationships needed to facilitate meaningful involvement. Co-designed projects do not just ‘happen’ but often require a long lead-in time to identify key stakeholders and partners, build relationships and establish credibility. This can be particularly challenging when working with traditionally under-served groups who may view service providers with suspicion or frustration [37, 38].

The importance of relationship building is well-recognised in the literature on co-design. Ní Shé and Harrison note that this relationship work is often “invisible” yet crucial for developing trust and establishing mutual aims and values between participants [39]. O’Mara Eves and colleagues’ review on community engagement similarly notes the quality of relationships between stakeholders (community members and service providers) impacts on the success of co-designed programmes [8]. Lowe and Plimmer talk about the importance of “being human” within the co-design process, “building empathy between people so that they can form effective relationships, understanding the strengths that each person brings, and deliberately working to create trust between people” [36].

As demonstrated by our case studies, there are different ways to build these relationships: partnering with well-respected local voluntary organisations (Men’s Project); commissioning private companies (Active People); or using in-house resources (Healthy Choices, FitnFun). Each of these options has their own advantages and disadvantages – the appropriate choice for a project will need to be considered in relation to the local context, the resources and skills available, and the ability to negotiate a shared vision of co-design between partners. Regardless of approach, co-design partners need to consider carefully whether they are truly reaching the people they most need to work with to address inequalities.

### Create supportive organisational cultures

Local authorities and NHS organisations often provide the funds for health-related co-design, yet our findings suggest that prevailing cultures and practices around funding and commissioning may at times hinder co-design. As our case studies identify, funding for co-design can be short-term and unpredictable. A recent review by Smith and colleagues identified the importance of “prolonged involvement with service users” as a pre-condition for co-design, yet acknowledged current funding approaches often hinder meaningful involvement [26]. In particular, they note that lack of funding in the early stages of a project can inhibit the establishment of meaningful relationships between service providers and community members, which appears so essential to the success of co-design [26]. Similarly, Halvorsrud et al. conclude that without sufficient time and resources, community involvement will remain tokenistic [13].

Our findings suggest three consequences of this unstable funding landscape. First, a proliferation of dis-jointed and short-lived projects which fail to address the systematic nature of the problem. Second, an emphasis on lower levels of community involvement (e.g. consultation, engagement) which are more easily achieved with limited resources. And third, potential harm to communities when budgets are unexpectedly cut. To counter these barriers to meaningful co-design, funders and commissioners should think carefully about the sustainability of the work and how to maintain long-term relationships with communities.

Traditional commissioning approaches with local authorities and Integrated Care Boards may not fit easily with co-design, with some questioning whether current institutional systems and practices can fully support the ideals of co-design [40]. Commissioning practices which focus on “specific objectives” and pre-determined outputs… measured by a limited set of indicators may conflict with the “messiness” of co-design [38]. Co-design focuses on what matters most to the people who use the services in order to re-align health services with community priorities [17].

Commissioning for true co-design therefore involves levels of uncertainty which, as noted by our participants, often sits uncomfortably with traditional commissioning approaches. Co-design is unlikely to flourish in a risk averse culture [15]. Within public service design, ambiguity is more often associated with “the risk of failure rather than innovation” [39]. Our findings suggest commissioners must get comfortable with this uncertainty and cultivate a “risk aware” rather than “risk averse” culture in order to realise the potential of co-design [41].

Creating a supportive culture for co-design may require funding organisations to view commissioning in a different way. Hart suggests traditional approaches to commissioning may be the “enemy of coproduction,” hindering any meaningful focus on what really matters to people [42]. Yet, the work of Strokosch & Osborne [43] is valuable here in suggesting (in words similar to one of our participants) that commissioners should focus on designing *for* service, rather than *the* service. This may mean moving from a narrow focus on purchasing services to deliver short-term ‘outcomes’, to an emphasis on producing longer-term ‘value’ as an emergent property of a complex system [44, 45]. Within the Men’s Project, for example, the ‘value’ arose from recognising the wider social and emotional contexts of the men’s lives, and creating a service landscape [43] which enabled on-going engagement, positively impacting many areas of their lives, including weight management. The study’s public contributors thought this was a particularly important point, feeling that many weight management services focus narrowly on diet and activity without recognising how weight is inseparable from all elements of people’s lives and is deeply influenced by mental and social wellbeing.

### Develop the evidence base

Finally, it is important to build the evidence base for co-design, to identify what works (and what doesn’t) in different contexts. As noted in several reviews, the evidence base for co-design remains under-developed because few studies report impact on outcomes [15, 16, 26, 46], with Smith and colleagues calling for more rigorous evaluation to help establish causal links [26]. Commissioners in our study were aware the evidence base for co-design was currently under-developed and were keen to see examples and learn from co-design conducted elsewhere. Yet evaluating co-design can be challenging. Co-design is inherently relational, contextual, messy and complex [47–49]. Effects are often subtle and longer-term [27]. Costs incurred in one area (for example, weight management) may produce positive (or negative) impacts in many others (for example, mental health) [47]. None of these factors lend themselves to standard forms of “scientific” evaluation [10, 48] which risk rendering the (positive and negative) effects of co-design invisible. Though some co-designed interventions may be amenable to formal testing through, for example, randomised controlled trials [50], in other cases a different approach may be needed. Theoretically-informed and rigorously-applied qualitative and mixed-methods approaches are likely to be able provide evidence of use to those wanting to commission co-design. The new framework [51, 52] for developing and evaluating complex interventions produced by the UK’s Medical Research Council is likely to be valuable here, particularly its focus on ‘theory-based’ and ‘systems’ evaluation perspectives. In addition, the Health CASCADE network is working to “strategically set the foundations for building evidence-based co-creation” in public health and has produced useful step-by-step guidance for those interested in this approach [53].

### Strengths and limitations

This study offers novel insights into co-designed weight management services, identifying key conditions required, as well as challenges to implementation. The case study approach allowed us to explore the wide range of activities that fall under the co-design umbrella, providing useful examples and insights for commissioners interested in this approach. However, not all case studies were able to fully implement their co-design activities. Consequently, only two projects had involved community members in the co-design and we were only able to recruit three community participants from one of these projects. We are therefore unlikely to have research data saturation within these community participant interviews. The perspective of local people involved in the co-design activities is therefore limited, resulting in a greater focus on commissioner/provider perspectives. It is also noted that all case studies were drawn from one region of the UK (including city, town and rural areas) though we believe our key considerations will be relevant in other countries and contexts. Finally, we also acknowledge that some co-design activities happened over a year before the interviews took place which may have affected people’s recollection.

## Conclusion

Co-design is an increasingly popular approach to designing health interventions. Drawing on four case studies of adult weight management services, we highlight both the potential benefits of, and barriers to, co-design. We present key considerations for those wanting to co-design health services, noting the importance of building strong relationships, creating supportive organisational cultures, and developing the evidence base.

### Electronic supplementary material

Below is the link to the electronic supplementary material.

Supplementary Material 1: Interview topic guide.

Supplementary Material 2: Coding framework.

## Data Availability

Data from this study are stored at https://data.bris.ac.uk/data/ and are available under ‘controlled access’. Requests for use will be referred to the University of Bristol Data Access Committee for approval before data can be shared under a data sharing agreement.

## Abbreviations

AP: Active People
C: Commissioner
CM: Community Member
FnF: FitnFun
HC: Healthy Choices
ICB: Integrated Care Board (Statutory NHS organisation responsible for commissioning and delivering services to meet the health needs of the local population.)
LA: Local Authority (Local government responsible for proving a wider range of services including public health provision.)
MP: Men’s Project
NHS: National Health Service
P: Provider
PPI: Patient and Public Involvement

## References

[1] NHS Digital, Health Survey for England, 2021 Part 1. 2022: Available from: https://digital.nhs.uk/data-and-information/publications/statistical/health-survey-for-england/2021.

[2] UK Government, Ethnicity facts and igures: Overweight adults. 2023: Available from: https://www.ethnicity-facts-figures.service.gov.uk/health/diet-and-exercise/overweight-adults/latest.

[3] Public Health England, Obesity and weight management for people with learning disabilities: guidance. 2020: Available from: https://www.gov.uk/government/publications/obesity-weight-management-and-people-with-learning-disabilities/obesity-and-weight-management-for-people-with-learning-disabilities-guidance.

[4] Department of Health & Social Care, Tackling obesity: empowering adults and children to live healthier lives. 2020: Available from: https://www.gov.uk/government/publications/tackling-obesity-government-strategy/tackling-obesity-empowering-adults-and-children-to-live-healthier-lives#empowering-everyone-with-the-right-information-to-make-healthier-choices.

[5] National Health Service, The NHS Long Term Plan. 2019: Available from: https://www.longtermplan.nhs.uk/wp-content/uploads/2019/08/nhs-long-term-plan-version-1.2.pdf.

[6] National Instuitute for Health and Care Excellence, Obesity: identification and management. Clinical Guideline. 2023: Available from www.nice.org/guidance/cg189. 36719951

[7] Birch JM (2022). A systematic review of inequalities in the uptake of, adherence to, and effectiveness of behavioral weight management interventions in adults. Obesity Reviews.

[8] O’Mara-Eves, A., et al., Community engagement to reduce inequalities in health: A systematic review, meta-analysis and economic analysis. Public Health Research, 2013;1(4). 25642563

[9] Fusco F, Marsilio M, Guglielmetti C (2020). Co-production in health policy and management: a comprehensive bibliometric review. BMC Health Services Research.

[10] McGill, B., et al. Co-produce, co-design, co-create, or co-construct—who does it and how is it done in chronic disease prevention? A scoping review in Healthcare, 2022. MDPI. 10.3390/healthcare10040647 PMC9029027 35455826

[11] Dudau A, Glennon R, Verschuere B (2019). Following the yellow brick road?(Dis) enchantment with co-design, co-production and value co-creation in public services. Public Management Review.

[12] Filipe A, Renedo A, Marston C (2017). The co-production of what? Knowledge, values, and social relations in health care. PLoS Biology.

[13] Halvorsrud K (2021). Identifying evidence of effectiveness in the co-creation of research: a systematic review and meta-analysis of the international healthcare literature. Journal of Public Health.

[14] Greenhalgh T (2016). Achieving research impact through co-creation in community‐based health services: literature review and case study. The Milbank Quarterly.

[15] Voorberg WH, Bekkers VJ, Tummers LG (2015). A systematic review of co-creation and co-production: embarking on the social innovation journey. Public Management Review.

[16] Clarke D (2017). What outcomes are associated with developing and implementing co-produced interventions in acute healthcare settings? A rapid evidence synthesis. BMJ open.

[17] Morales-Garzón S (2023). Addressing Health disparities through Community participation: a scoping review of Co-creation in Public Health. Healthcare.

[18] Talevski J (2023). Use of co-design methodology in the development of cardiovascular disease secondary prevention interventions: a scoping review. Health Expectations.

[19] Radl-Karimi C (2020). Under what circumstances can immigrant patients and healthcare professionals co-produce health? - an interpretive scoping review. International Journal of Qualitative Studies on Health and Well-being.

[20] Moore, T., et al., Methods used in co-produced behavioural weight management programmes for adults with overweight and obesity: A scoping review. 2023: Available from https://osf.io/qd6x8/?view_only=603057e7f61848c9aae18dcbf2592a49.

[21] Goff LM (2019). Healthy eating and active lifestyles for diabetes (HEAL-D): study protocol for the design and feasibility trial, with process evaluation, of a culturally tailored diabetes self-management programme for african-caribbean communities. BMJ open.

[22] Gray CM (2013). Football fans in training: the development and optimization of an intervention delivered through professional sports clubs to help men lose weight, become more active and adopt healthier eating habits. BMC Public Health.

[23] Coupe N, Cotterill S, Peters S (2022). Enhancing community weight loss groups in a low socioeconomic status area: application of the COM-B model and Behaviour Change Wheel. Health Expectations.

[24] Vargas C (2022). Co-creation, co-design, co-production for public health: a perspective on definition and distinctions. Public Health Research & Practice.

[25] Masterson D (2022). Mapping definitions of co-production and co-design in health and social care: a systematic scoping review providing lessons for the future. Health Expectations.

[26] Smith H (2022). Co-production practice and future research priorities in United Kingdom-Funded applied health research: a scoping review. Health Research Policy and Systems.

[27] Williams, O., et al., Is co-production just really good PPI? Making sense of patient and public involvement and co-production networks, in Decentring health and care networks: Reshaping the organization and delivery of healthcare, M. Bevir and J. Waring, Editors. Palgrave Macmillan Cham. 2020:213–237.

[28] Locock, L. and A. Boaz, Drawing straight lines along blurred boundaries: Qualitative research, patient and public involvement in medical research, co-production and co-design. Evidence & Policy, 2019;15(3):409–421.

[29] Lewis J, Issues Design, Ritchie J, Lewis J (2003). Qualitative research practice.

[30] Terry G, Willig C (2017). Thematic analysis. The SAGE handbook of qualitative research in psychology.

[31] Clarke V, Braun V (2013). Successful qualitative research: a practical guide for beginners. Successful qualitative research.

[32] Robertson C (2016). Should weight loss and maintenance programmes be designed differently for men? A systematic review of long-term randomised controlled trials presenting data for men and women: the ROMEO project. Obesity Research & Clinical Practice.

[33] de Souza P, Ciclitira KE (2005). Men and dieting: a qualitative analysis. Journal of Health Psychology.

[34] Elliott M, Gillison F, Barnett J (2020). Exploring the influences on men’s engagement with weight loss services: a qualitative study. BMC Public Health.

[35] Cottam, H. and C. Leadbeater, Red Paper 01 Health: Co-creating services. 2004: Available from: https://www.designcouncil.org.uk/fileadmin/uploads/dc/Documents/red-paper-health.pdf.

[36] National Co-production Advisory Group, *Ladder of co-production*. 2021: Available from: https://www.thinklocalactpersonal.org.uk/_assets/COPRODUCTION/Ladder-of-coproduction.pdf.

[37] Bopp, M., et al., Engaging community partners to develop a culturally relevant resource guide for physical activity and nutrition. Ethnicity & Disease, 2012;22(2):231–238. 22764648

[38] Moll S (2020). Are you really doing ‘codesign’? Critical reflections when working with vulnerable populations. BMJ open.

[39] Ní Shé É, Harrison R (2021). Mitigating unintended consequences of co-design in health care. Health Expectations.

[40] Madden M (2020). Producing co-production: reflections on the development of a complex intervention. Health Expectations.

[41] Social Care Institute for Excellence, Co-production: what it is and how to do it. 2022: Available from: https://www.scie.org.uk/co-production/what-how.

[42] Hart F (2022). Is commissioning the enemy of co-production?. Perspectives in Public Health.

[43] Strokosch, K. and S.P. Osborne, Design of services or designing for service? The application of design methodology in public service settings. Policy & Politics, 2023;51(2):231–249.

[44] Lowe T, Plimmer D (2019). Exploring the New World: practical insights for funding, commissioning and managing in complexity.

[45] Knight AD (2017). A whole new world: funding and commissioning in complexity.

[46] Slattery P, Saeri AK, Bragge P (2020). Research co-design in health: a rapid overview of reviews. Health Research Policy and Systems.

[47] Boyle, D., et al., Right here, right now. Taking co-production into the mainstream. 2010, New Economics Foundation: London.

[48] Durose C (2017). Generating ‘good enough’evidence for co-production. Evidence & Policy.

[49] Donetto S (2015). Experience-based co-design and healthcare improvement: realizing participatory design in the public sector. The Design Journal.

[50] Leask CF (2019). Framework, principles and recommendations for utilising participatory methodologies in the co-creation and evaluation of public health interventions. Research Involvement and Engagement.

[51] Skivington K (2021). Framework for the development and evaluation of complex interventions: gap analysis, workshop and consultation-informed update. Health Technol Assess.

[52] Skivington, K., et al., A new framework for developing and evaluating complex interventions: update of Medical Research Council guidance BMJ, 2021;374:n2061. 10.1136/bmj.n2061 PMC8482308 34593508

[53] Agnello, D. and D. Longworth, A Draft Evidence-based Co-Creation Guideline: PRODUCES+ (Version 1). Zenodo. 2022: Available from: 10.5281/zenodo.8379784.

