# Supplementary material for: Co-designing adult weight management services: a qualitative study exploring barriers, facilitators, and considerations for future commissioning
Source: BMC Public Health. 2024 Mar 12;24:778. doi: 10.1186/s12889-024-18031-w (PMC10935989; doi:10.1186/s12889-024-18031-w)
Supplement: Supplementary file 1 — Supplementary Material 1: Interview topic guide. [file 12889_2024_18031_MOESM1_ESM.docx]

# Additional file 1

## Topic guide for commissioners and providers

| ***Introduction*** | |
| --- | --- |
| ***Professional role and experience*** | |
| Tell me a bit about your role and your involvement with weight management? | |
| ***Overview of weight management project*** | |
| First, can you give me a brief summary of the [NAME] programme | - e.g. target population, what it involved, how long, how delivered? |
| How have you been involved in the programme? | - What was your role? |
| Can you give me a bit of background context to how [name of programme] came about in [name of Local Authority/third sector organisation] | - Why was this new programme needed? (Probe: under-served populations?) - How does this compare with other weight management approaches that you are aware of, locally or nationally? |
| ***Reasons for co-designed weight management services*** | |
| Why did you choose to use a co-design approach for this project? | - Advantages over traditional services? - What benefits did you hope for? |
| Are your views on codesign shared across your Local Authority/organisation? | - Is there a vision for co-designed weight management interventions? - Written down/available online? |
| ***Co-design process*** | |
| Walk me through the process that was used to co-design [name of programme]? | - Who was involved? - What activities/methods/approaches were used? - What level of involvement (informed, consulted, collaborating, leading) - Who led the process? - What stage did this take place? - How were differing ideas/opinions managed? - Who had the deciding vote on ideas to be included? - How were decisions made about whether and how to make necessary adjustments to the programme after it had been launched? And by whom? |
| ***Participant Engagement*** | |
| How did you find people’s engagement with the [programme]? | - Was this different from weight management programmes that haven’t been co-designed? |
| Did you notice any difference in what people got out of the programme, compared to traditional weight management programmes you have been involved with? | |
| ***Reflection on co-designed weight management services*** | |
| Has the codesigned project achieved what you’d hoped it would? | - Meeting its aims? - Does the final WM programme meet your expectations as a commissioner? |
| What were the biggest benefits of carrying out the co-designed process for [name of programme]? | |
| What were/are the biggest challenges of the co-design approach? | |
| Were there any other contextual or wider factors that influenced the co-design or implementation of [name of programme]? | |
| Are/were all the people who needed to be involved in the co-design process, in this case, actually involved? | - Why? Why not? - How could this be addressed? |
| Do you feel that co-designing [name of programme] was an efficient use of time and money? | - How could this have been made more efficient? |
| What are your key lessons learned from the co-design process? | |
| If other areas were going to use a co-design approach to weight management services, what advice would you give. | |
| ***Any other issues*** | |
| - Any other issues you would like to raise? - Is there anything important I have not asked you about? - Do you have any recommendations on who else might be useful to speak to about [name of programme] or co-designed weight management services in general? | |

## Topic guide for community members involved in co-design activities

|  | |
| --- | --- |
| ***Can you tell me a little bit about how you got involved in designing [name of programme]?*** | - *How did you hear about it?* - *Why did you want to get involved?* - *Had you done anything like this before?* |
| ***Tell me about your role in designing the programme.*** | - *What tasks were involved?* - *Who led the tasks/discussion?* - *How many others were involved?* - *What sorts of things were you asked about?* - *What sort of feedback did you give on the programme?* - *Do you know what changes were made as a result of your feedback?* |
| ***How did you find the process of helping designing the programme?*** | - *Did you feel your ideas were listened to?* - *What worked well?* - *What was more challenging?* - *What have you learnt from participating in the process?* |
| ***Did you take part in the final health programme?*** | - *What did you think of the programme?* - *Was it different to other health programmes you’ve been involved with?* |
| ***Thinking more generally, what do you think about involving local people in designing local health services like [name of programme]?*** | - *What benefits are there from involving local people? (For the programme? For you personally?)* - *What challenges are there to involving local people?* - *Were the right people involved in designing this programme?* |
| ***Finally, what advice would you give to NHS/Local council staff who want to involve local people in planning their services?*** | |
| ***Thank you for taking part in this interview. Are there any other issues you want to raise? Is there anything important I have not asked you about?*** | |
