# Supplementary material for: Co-designing adult weight management services: a qualitative study exploring barriers, facilitators, and considerations for future commissioning
Source: BMC Public Health. 2024 Mar 12;24:778. doi: 10.1186/s12889-024-18031-w (PMC10935989; doi:10.1186/s12889-024-18031-w)
Supplement: Supplementary file 2 — Supplementary Material 2: Coding framework. [file 12889_2024_18031_MOESM2_ESM.docx]

# Co-design Weight Management Services Coding Framework

Context or Background

Co-design Process Description

Why a co-design approach was needed

Description of final programme

Engagement

Future plans and sustainability

Definitions – co, design, co-production, co-creation

Co-design as culture

Benefits of co-design

Barriers to co-design

Time

Relationship building

Risks and harms

Uncertainty

Commissioning

Evidence and Evaluation

Working with the right people

Efficient use of resources

Learning for others
